# Integrated One-Health analysis of antimicrobial resistance, biofilm formation, and genetic relatedness of *Enterococcus* spp. at the human-food interface

**DOI:** 10.1038/s41598-026-63849-6

**Published:** 2026-08-05

**Authors:** Heba A. Ahmed, Tamer M. El Feky, Marwa Shalaby, Mahmoud E. Elsotohy, Rasha A. Mohsen, Alshymaa A. Ahmed

**Affiliations:** 1 Department of Zoonoses, Faculty of Veterinary Medicine, Zagazig University, Zagazig City, 44511 Sharkia Governorate Egypt; 2 Department of Bacteriology, Animal Health Research Institute, Mansoura Branch, Agriculture Research Center (ARC), Mansoura, 12618 Egypt; 3 Department of Clinical Pathology, Faculty of Medicine, Zagazig University, Zagazig City, Sharkia Governorate Egypt

**Keywords:** *Enterococcus* species, MDR, Biofilm formation, RAPD-PCR, One Health, Microbiology, Molecular biology

## Abstract

*Enterococcus* species are opportunistic pathogens and reservoirs of antimicrobial resistance (AMR) that can be transmitted through food chain. This study investigated *Enterococcus faecalis* and *Enterococcus faecium* isolated from retail meat, workers and consumers within a One Health framework. A total of 250 samples yielded 50 (20%) isolates, which were analyzed for antimicrobial resistance, virulence genes, biofilm formation, and RAPD-PCR genotyping. This study provides an integrated One Health assessment linking food contamination, occupational exposure, and human colonization. Beef meat showed the highest isolation rate, and *E. faecium* predominated among consumers. High resistance rates were observed for rifampin (94%, 47/50), erythromycin, and tetracycline (88%, 44/50, each), while vancomycin resistance was low (8%, 4/50). Multidrug resistance was observed in 88% of isolates, and MAR index ranged from 0.07 to 0.86 (average = 0.43). Biofilm formation occurred in 68% (34/50) of isolates, with moderate strength predominated. Resistance genes were widely distributed, particularly *tet*L (88%, 44/50), while *esp* (84%, 42/50) and *gel*E (68%, 34/50) were the most prevalent virulence genes. RAPD-PCR revealed five clusters with moderate discriminatory power (D = 0.729), demonstrating genetic relatedness across sources. Importantly, the co-occurrence of resistance, virulence, and biofilm traits across genetically related isolates highlights the possibility of the food chain as a potential transmission interface. These findings highlight the role of *Enterococcus* species in AMR dissemination within a One-Health framework.

## Introduction

*Enterococci* are Gram-positive, facultative anaerobic cocci that naturally inhabit the gastrointestinal tract of humans and animals^1^. Although members of the genus *Enterococcus* constitute part of the human intestinal microbiota, several species have emerged as important opportunistic pathogens in recent decades, particularly among hospitalized and immunocompromised patients^2^. *Enterococcus faecalis* and *E. faecium* are the most clinically significant species, accounting for more than 90% of *Enterococcus* isolates recovered from human patients^3^. These species are widely distributed in various environments, such as the gastrointestinal tracts of humans and animals, as well as in soil, water, and food products^3,4^. In healthcare settings, they are associated with various infections, such as urinary tract infections, endocarditis, and wound infections, particularly in immunocompromised and hospitalized patients^5,6^.

*E. faecalis* and *E. faecium* ability to persist on food-producing animals highlights their zoonotic potential and the possibility of foodborne disease transmission, posing a significant public health concern^7^. Since 1990s, several studies have suggested the zoonotic transmission of antimicrobial-resistant enterococci. Vancomycin-resistant enterococci (VRE) have emerged as nosocomial pathogens worldwide, however, reservoirs of VRE have also been identified in community settings and in food-producing animals. The use of vancomycin-analogue avoparcin as a growth promoter in livestock has been strongly associated with the emergence of VRE in poultry and pigs^8^. Moreover, the occurrence of VRE in food animals and meat products has been correlated with the occurrence of VRE in human stool samples^9^. There is a high risk that animal VRE could be transmitted through the food chain since enterococci are particularly resistant to disinfectants, heat, and other decontamination procedures^10^.

Animal-associated enterococci may be transmitted to humans through direct contact with animals or contaminated environments, or indirectly through consumption of contaminated food of animal origin and vegetables fertilized with animal manure^7^. *E. faecium* and *E. faecalis* frequently contaminate raw meat at concentrations of 10^2^–10^4^ colony forming units (CFU) per gram^10^. Among food products, poultry meat has been identified as an important source for transmission of antimicrobial-resistant enterococci to humans, mainly due to cross-contamination during slaughter and processing, as well as high consumption rates^7,11^. Evidence supporting zoonotic transmission also includes the clonal relatedness between isolates recovered from poultry meat and those from human stool samples^12^.

Retail meat handlers may also contribute to the dissemination of enterococci along the food chain. This is a result of insufficient personal hygienic measures, especially hand washing because enterococci inhabit intestinal tract and then transmitted through fecal contamination^13^.

Antimicrobial resistance in enterococci represents a growing public health concern. Both *E. faecalis* and *E. faecium* exhibit intrinsic resistance to several commonly used antimicrobials, and some strains have acquired additional resistance mechanisms, resulting in the emergence of multidrug-resistant (MDR) enterococci^14^.

Previous studies have reported MDR *Enterococcus* strains harboring various determinants in food producing animals, such as poultry and cattle^15,16^. In addition, biofilm formation represents an important factor contributing to enterococcal antimicrobial resistance^17^. Biofilms are structured microbial communities embedded within a self-produced extracellular matrix that enhances bacterial survival under diverse conditions^18^. The ability of enterococci to form biofilms on food processing surfaces protects them from sanitizers and facilitates long-term contamination. Moreover, biofilms may promote horizontal transfer of antimicrobial resistance genes among bacterial population^19^.

Despite increasing reports on antimicrobial-resistant *Enterococcus* species from food and clinical sources, most studies have investigated these reservoirs separately, with limited integration between phenotypic resistance, virulence determinants, and biofilm formation across interconnected human and food isolates. Moreover, studies simultaneously linking these characteristics across interconnected human and food sources within the same geographical setting remain scarce. Therefore, this study aimed to provide an integrated One Health analysis of *E. faecalis* and *E. faecium* isolated from retail meat, workers, and consumers within the same geographical setting, combining phenotypic characterization, resistance and virulence gene profiling, biofilm assessment, and genotyping to better understand potential transmission pathways and co-selection.

## Materials and methods

### Sample collection

A total of 250 samples were collected during the study, including 150 retail meat samples (chicken, beef, and camel meat, 50, each) and 100 human stool swabs obtained from retail market workers and consumers (50, each) in Zagazig City, Sharkia Governorate, Egypt. Chicken and beef samples were obtained from 10 retail shops, while camel meat were collected from the five shops that sold camel meat at the study area. The sample size was determined based on convenience sampling considering accessibility of sampling sites, and participant availability while ensuring representation of different sources within the study area. Sampling was conducted aseptically in sterile plastic bags during the period from April to June 2025. Human stool samples were collected from workers at the retail markets, and from consumers within the same study area using sterile collection cups. A sterile swab was taken from each stool sample and inserted in a collection tube containing buffered peptone water. Samples and swabs were transported in an icebox (4 °C) to the laboratory and processed within 12–24 h.

### Ethics approval and consent to participate

The study was approved by Zagazig University Institutional Animal Care and Use Committee (ZU-IACUC) with approval number ZU-IACUC/2/F/170/2025. Ethical approval for procedures involving human participants was obtained from the Research Ethical Committee of Faculty of Medicine, Zagazig University. All procedures involving human participants were conducted in accordance with the principles of the Declaration of Helsinki. Written informed consent was obtained from the retail market workers and consumers for their participation in this study.

### Samples preparation and isolation

Sample preparation was performed according to the guidelines of ISO 6887-2^20^. For each meat sample, 25 g were aseptically weighed and homogenized with 225 mL of 0.1% Buffered Peptone Water (BPW; HIMEDIA, M614-500G, India) using a stomacher (Colworth 400, Seward Ltd., UK) for 2.5 min at room temperature (25 °C). Subsequently, 1 mL of this homogenate was transferred into a sterile test tube containing 9 mL of 0.1% BPW and incubated at 37 ± 0.5 °C for 24 h. From each sample, 100 µl was inoculated onto bile esculin azide agar (BEA; HiMedia, M340, India), and incubated aerobically at 37 ± 0.5 °C for 24 h. Colonies showing the typical morphology of enterococci, small (approximately 1 mm in diameter), greyish-white with surrounding black or brown discoloration due to esculin hydrolysis, were considered presumptive *Enterococcus* species according to ISO 7899-2^21^.

Presumptive colonies were subsequently sub-cultured in brain heart infusion broth (BHI; Oxoid, CM1135, UK) and incubated at 37 °C for 24 h to obtain purified cultures.

### Identification of *Enterococcus* isolates

Presumptive *Enterococcus* isolates were initially identified using conventional biochemical tests, including bile esculin hydrolysis, growth in 6.5% NaCl, arabinose fermentation, and pyruvate utilization following standard procedures^22^.

Genomic DNA was extracted from biochemically confirmed isolates using the QIAamp DNA Mini Kit (Qiagen GmbH, Hilden, Germany, Cat. No. 51304) according to the manufacturer’s instructions. Molecular confirmation of *Enterococcus* spp. was carried out by the amplification of 16 S rRNA gene using specific primers^23^. Further identification at the species level was conducted by PCR amplification of species-specific genes, including the16S rRNA gene specific for *E. faecalis*^24^ and the *sod*A gene specific for *E. faecium*^25^.

### Antimicrobial susceptibility testing and detection of resistance genes

Antimicrobial susceptibility of *Enterococcus* isolates was evaluated against 14 antibiotics belonging to nine antimicrobial classes using the Kirby-Bauer disk diffusion method on Mueller–Hinton agar (Himedia, Mumbai, India) following the Clinical and Laboratory Standards Institute (CLSI) M100 Performance Standards for Antimicrobial Susceptibility Testing^26^. The tested antibiotics included Penicillin (P, 10 U); Ampicillin (AM, 10 µg); Erythromycin (E, 15 µg); Tetracycline (TE, 30 µg); Doxycycline (DO, 30 µg); Ciprofloxacin (CIP, 5 µg); Levofloxacin (LEV, 5 µg); norfloxacin (NOR, 10 µg); Gatifloxacin (GAT, 5 µg); Nitrofurantoin (F, 300 µg); Rifampin (RD, 5 µg); Chloramphenicol (C, 30 µg); Vancomycin (VAN, 30 µg); and Trimethoprim/sulfamethoxazole (SXT, 1.25/23.75 µg).

Briefly, bacterial suspensions equivalent to 0.5 McFarland turbidity standard were prepared and inoculated onto Mueller–Hinton agar plates. Antibiotic discs were placed on the inoculated agar surface, and the plates were incubated at 37 °C for 24 h. Inhibition zone diameter were measured and interpreted according to CLSI breakpoints^26^. For vancomycin susceptibility testing, the broth microdilution method was used to determine the minimum inhibitory concentration (MIC). *E. coli* ATCC 25,922 was used as the quality control strain.

The multiple antimicrobial resistance index (MAR) was calculated as the ratio of the number of antimicrobial agents to which *Enterococcus* isolates displayed resistance to the total number of antimicrobial agents tested^27^. Multidrug resistance (MDR) was defined as resistance to at least one agent from three or more antimicrobial classes, while extensively drug resistant (XDR) isolates were defined as isolates resistant to at least one agent in all but two or fewer antimicrobial categories^28^. Antimicrobial resistance genes *van*A^29^, *van*B^30^, *tet*L, *tet*M, *tet*S, *pbp*5^31^, *bla*TEM^32^ were amplified as previously described.

### Biofilm formation ability of *Enterococcus* isolates

The biofilm-forming ability of *E. faecalis* and *E. faecium* isolates was evaluated using 96 wells microtiter plates method^33^. The optical density (OD) was determined using an ELISA reader (model: sunrise R4, serial no: 610000079) at wavelength of 620 nm (OD_620_ nm) after subtracting the absorbance of the negative control. All experiments were performed in triplicates, and the results are represented as mean ± standard deviation (SD). The cut off value (ODc) was calculated by the formula: ODc= Average OD of negative control + (3x standard deviation of negative control). The mean OD value obtained from the media control well was subtracted from all test OD values (Biofilm OD = OD1 – ODc). The isolates were classified to four categories according to Saxena, et al.^34^:

$${\mathrm{Non}} - {\text{biofilm producer OD}}\, \le \,{\mathrm{ODc}}$$

$${\text{Weak biofilm producer}}\, = \,{\text{ODc }} < {\text{ OD}}\, \le \,{\mathrm{2}} \times {\mathrm{ODc}}$$

$${\text{Moderate biofilm producer}}\, = \,{\mathrm{2}} \times {\mathrm{ODc}}\, < \,{\mathrm{OD}}\, \le \,{\mathrm{4}} \times {\mathrm{ODc}}$$

$${\text{Strong biofilm producer}}\, = \,{\mathrm{4}} \times {\mathrm{ODc}}\, < \,{\mathrm{OD}}$$

### Virulence gene profiling of *E. faecalis* and *E. faecium* isolates

Virulence-associated genes were detected using SYBR Green real time PCR (qPCR). The investigated genes included gelatinase (*gel*E), enterococcal surface protein (*esp*) and cytolysin activator *(cyl*A, *cyl*B and *cyl*M) as previously detailed^31^. Moreover, aggregation substance (*asa*), *E. faecalis* antigen A gene (*efa*A), and adhesion of collagen from *E. faecalis* (*ace*) genes were also investigated^35^. Real time PCR was performed using primer sets (Alpha DNA, Canada) specific for each gene. SYBR Green I fluorogenic signal was collected and the cycle threshold (CT) of the reactions was detected by MJ OpticonMonitor™ Analysis software version 3.1 (Bio-Rad). Each isolate was tested in duplicate, positive and negative controls were included in all PCR runs. Additionally, no template controls (NTCs) were used for each primer set.

### RAPD-PCR genotyping of *Enterococcus* isolates

The genetic relatedness of *E. faecalis* and *E. faecium* isolates from different sources was evaluated by RAPD-PCR using specific primers, AP4 (5 TCA CGC TGC A 3) and ERIC1R (5 ATG TAA GCT CCT GGG GAT TCA C 3) as described previously^36^.

The RAPD fingerprinting data were transformed into a binary code depending on the presence (1) or absence (0) of each band. Cluster analysis and dendrogram construction were performed with the stats package in R-software version 4.3.3^37^ using binary/Jaccard distance^38^, and the unweighted pair group method with arithmetic average (UPGMA) method. The discriminatory power of RAPD-PCR was measured using the Simpson’s index of diversity (*D*), which represents the probability that two unrelated strains randomly selected from a population will be assigned to a different type by the typing method. A *D* value greater than 0.9 indicates high discriminatory ability^39^.

### Statistical analysis

Statistical analysis was performed using SPSS software version 26 (IBM Corp, Armonk, NY, USA) and R software version 4.3.3^37^(https://www.r-project.org/). The Chi-square test was used to study the variations in the prevalence of *Enterococcus* isolates from different sources, and to assess the differences in the antimicrobial resistance patterns of the recovered isolates from various sources, and also the existence of various tested genes among the investigated isolates from various origins. A structured questionnaire was used to collect demographic and epidemiological data from workers and consumers. The investigated variables included age, gender, education level, residence, and diarrhea at the time of sampling. These variables were selected based on their potential association with exposure to antimicrobial-resistant foodborne bacteria as reported in previous epidemiological studies^40,41^. The presence or absence of *Enterococcus* species was considered the outcome variable. Binary logistic regression model was used to determine the influence of the predictor variables on the prevalence of *Enterococcus* isolates in workers and consumers. Random forest classification was utilized to identify the most significant variables for predicting *Enterococcus* prevalence utilizing the randomForest package^42^ in R. Before correlation analyses, variables were tested for normality using Q–Q plot. Pearson correlation (r) was estimated and visualized using the Hmisc package^43^ in R software version 4.3.3. Stacked bar plots, where sub-columns are calculated as a part of the total column, were used for visualization of the prevalence of *Enterococcus* from different sources, resistance to various antimicrobial agents and classes, distribution of resistance, and virulence genes, and differences in biofilm production abilities of *Enterococcus* isolates using the ggplot^44^ package in R software version 4.3.3. The p-values were considered statistically significant if they were less than 0.05. All graphs were generated by R using ggplot^44^, pheatmap^45^, and factoextra^46^ packages.

## Results

### Prevalence of *Enterococcus* species in different sources

**Fig. 1:**
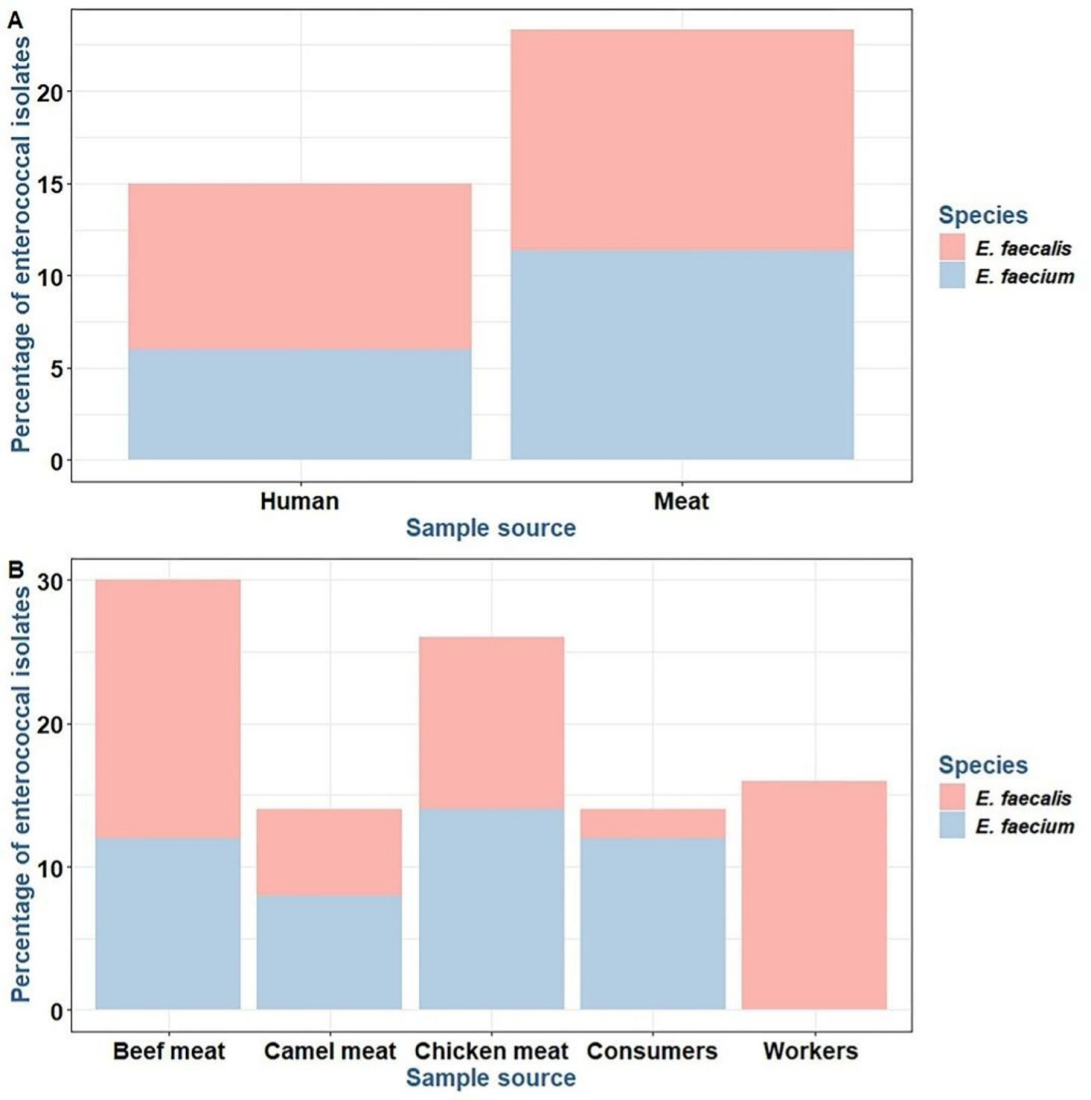
Stacked bar showing the prevalence of *Enterococcus* species among different sample sources.

A total of 50 *Enterococcus* isolates were recovered from meat and human samples (Table 1; Fig. 1), representing an overall prevalence of 20% (50/250). Among meat samples, the highest prevalence was recorded in beef meat (30%), followed by chicken (26) and camel meat (14%). Regarding species distribution in meat samples, *E. faecalis* and *E. faecium* were detected at rates of 12% and 11.3%, respectively. In human stool samples, 15 (15%) isolates were recovered, including 8 (16%) from retail workers and 7 (14%) from consumers. Only *E. faecalis* was recovered from retail workers (16%), while *E. faecium* was the most prevalent from consumers (12%).

**Table 1:** Prevalence of *Enterococcus* species among different samples.

Source (No.)	Total no. of Enterococcus isolates (%)^a^	*p*-value^b^	No. of *Enterococcus* spp. (%)^a^		*p*-value^b^
			*E. faecalis*	*E. faecium*	
**Meat (150)**	**35 (23.3)**		**18 (12)**	**17 (11.3)**	1
Chicken meat (50)	13 (26)	0.145	6 (12)	7 (14)	0.775
Beef meat (50)	15 (30)		9 (18)	6 (12)	0.577
Camel meat (50)	7 (14)		3 (6)	4 (8)	0.715
**Human (100)**	**15 (15)**		**9 (9)**	**6 (6)**	0.593
Workers (50)	8 (16)	1	8 (16)	0	0.006**
Consumers (50)	7 (14)		1 (2)	6 (12)	0.06
***p*** **-value** ^c^	**0.146**		**0.536**	**0.184**	
**Total (250)**	50 (20**)**		27 (10.8)	23 (9.2)	

^a^ The isolation rate was calculated concerning the total number of the examined samples from each source.

^b^ *p*-value among *Enterococcus* isolates from each host.

^c^ *p*-value among total meat and human isolates.

**p < 0.01.

No statistically significant differences were observed in the prevalences of total *Enterococcus* spp., *E. faecalis*, and *E. faecium* isolated from total meat and human samples (*p* = 0.146, 0.536, and 0.184, respectively). Similarly, no significant differences were found in the prevalence of total *Enterococcus* spp. among various meat and human samples (*p* = 0.145, and 1, respectively). However, a statistically significant difference was observed in the prevalence of *E. faecalis* and *E. faecium* among human worker samples (*p* = 0.006).

### Risk factors associated with the prevalence of *Enterococcus* spp. in human samples

**Fig. 2:**
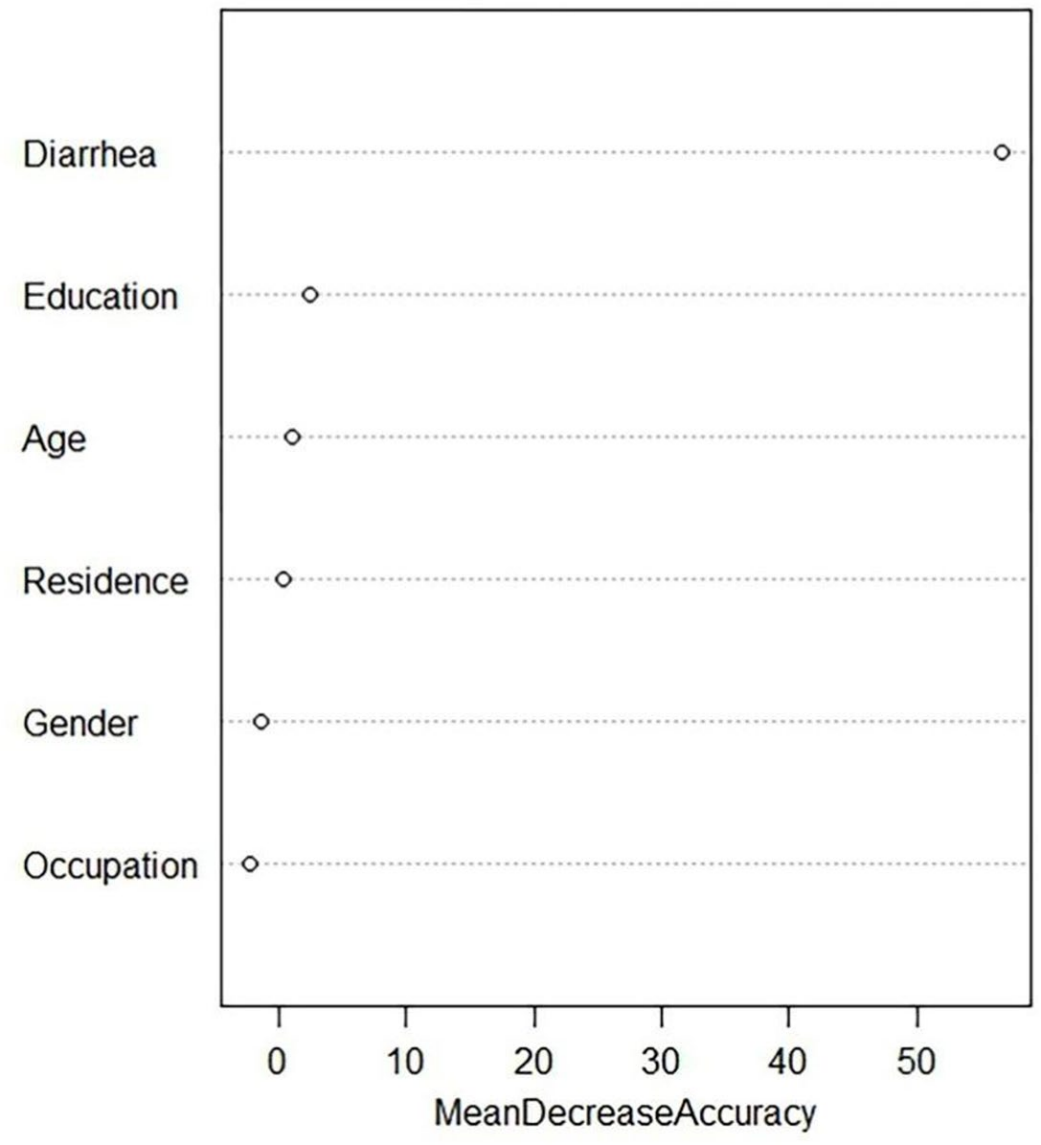
Random forest classification showing ranked importance for each factor in predicting the occurrence of *Enterococcus* spp. from human samples. The X-axis refers to the mean decrease in accuracy of the classification when the respective factor was removed.

The relative importance of different variables in predicting the occurrence of *Enterococcus* species in human samples, as determined by Random Forest model, is presented in Fig. 2. Among the investigated variables, diarrhea was identified as the most important variable in predicting the occurrence of human *Enterococcus* isolates, followed by education level and age.

Logistic regression analysis (Table 2) demonstrated that individuals without diarrhea had a significantly lower likelihood of *Enterococcus* occurrence compared with diarrheic individuals. No statistically significant associations were observed between *Enterococcus* prevalence and other variables, including age group, gender, education level, residence, and occupation (p > *0.05*).

**Table 2:** Logistic regression model of different variables associated with the prevalence of *Enterococcus* in humans.

Variables	Enterococcus			β-coefficient	SE (β)	*p*-value	OR (95% CI)
	Positive	Negative	Prevalence (%)				
Age							
≥ 41 (ref.)	11	55	16.7			1	
26–40	3	15	16.7	-17.91	20096.5	0.99	0 (0)
16–25	0	9	0	-17.92	13108.1	0.99	0 (0)
≤ 15	1	6	14.3	-0.113	1.38	0.94	0.893 (0.06–13.33)
Gender							
Male (ref.)	9	41	18				
Female	6	44	12	-17.99	20096.5	0.99	0 (0)
Education							
Educated (ref.)	3	14	17.6				
Elliterate	12	71	14.5	-0.529	1.51	0.73	0.589 (0.03–11.38)
Residence							
Rural (ref.)	11	67	14.1				
Urban	4	18	18.2	0.097	1.23	0.94	1.1 (0.098–12.33)
Occupation							
Consumers (ref.)	7	46	13.2				
Workers	8	39	17	-18.9	20096.5	0.99	0 (0)
Diarrhea							
Positive (ref.)	13	2	86.7				
Negative	2	83	2.4	-5.69	1.21	< 0.0001***	0.003 (0-0.036)

β: Regression coefficient; SE: standard error; OR: odds ratio; CI: confidence interval; ref: reference; ***p < 0.0001.

### Antimicrobial resistance profile of *E. faecalis* and *E. faecium* from different sources

**Fig. 3:**
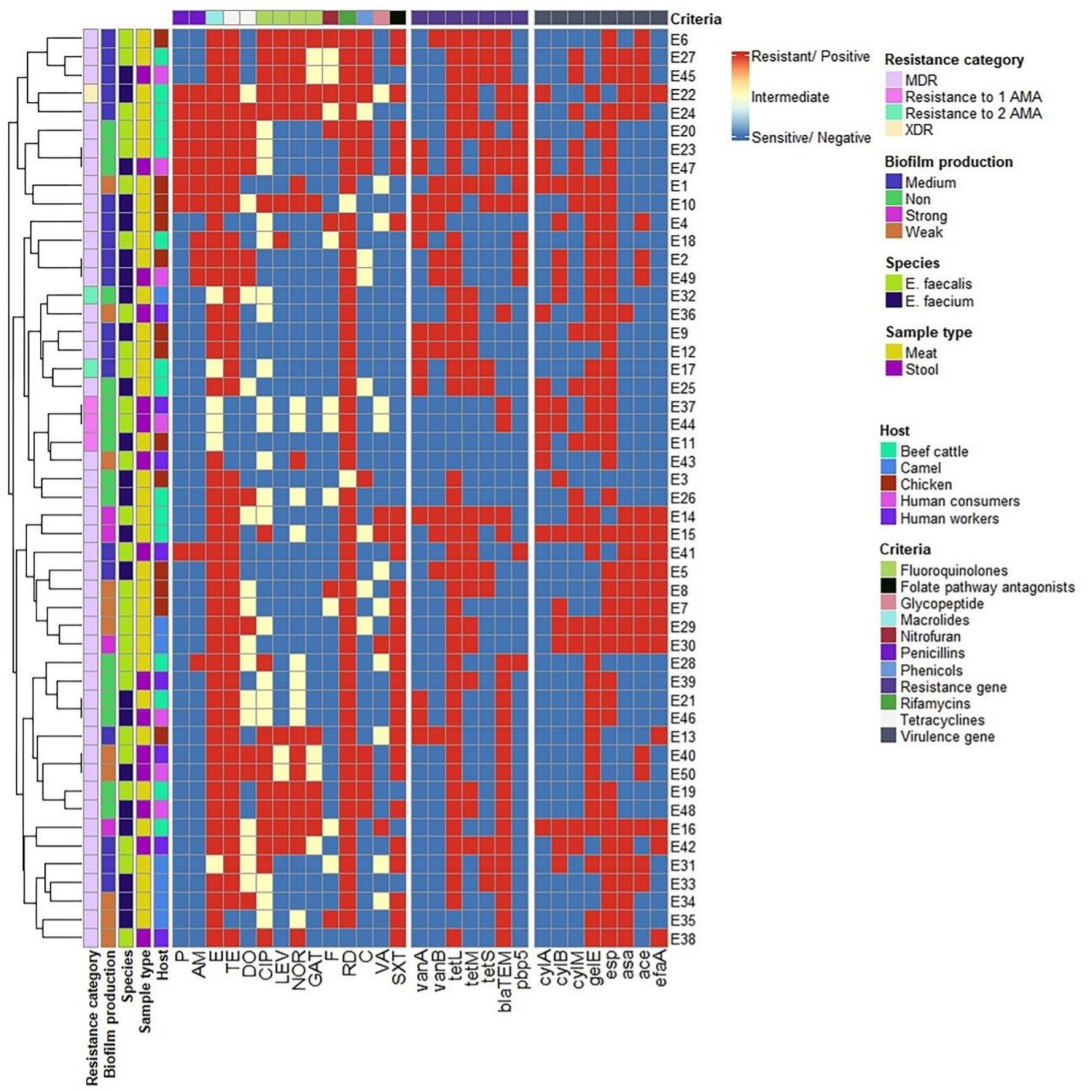
Hierarchical clustering heatmap showing the overall distribution of the investigated Enterococcus isolates based on the phenotypic antimicrobial resistance pattern, resistance genes, and virulence genes. Different host, sample sources, resistance categories, isolate biofilm-forming ability, and antimicrobial classes are color-coded on the right of the heatmap.

**Fig. 4:**
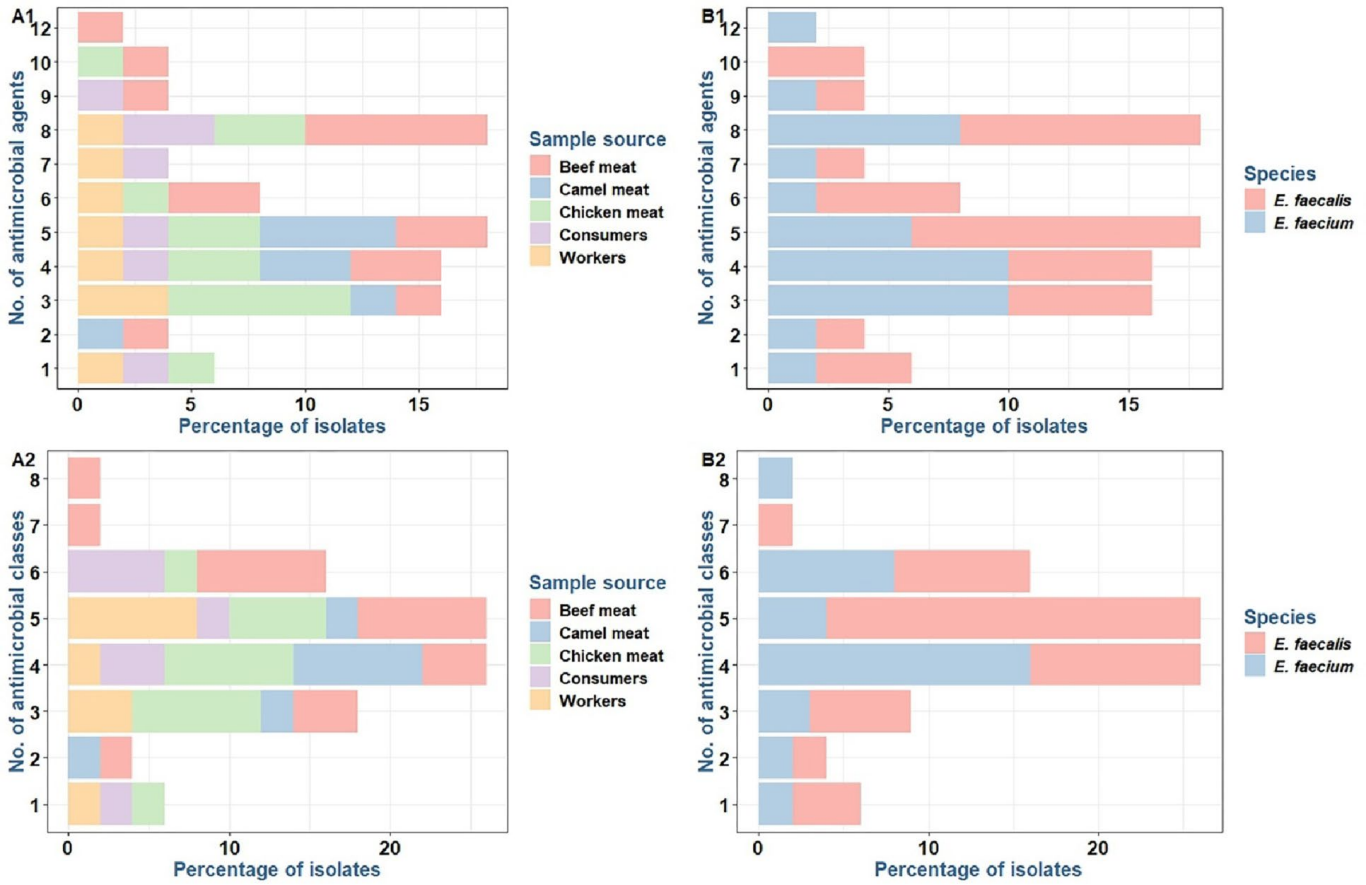
Stacked bar showing the resistance to various antimicrobial agents (**A1**) and classes (**A2**) in *Enterococcus* isolates obtained from different sources (**A1**, **A2**), and different species (**B1**, **B2**). ^*^ The frequency was calculated concerning the total number of the examined isolates (*n* = 50) for each parameter, and subcolumns are calculated as a part of the total column.

The antimicrobial resistance patterns of *E. faecalis* (*n* = 27) and *E. faecium* (*n* = 23) isolates were evaluated against 14 antimicrobial agents, and the results are summarized in Tables 3 and 4; Figs. 3 and 4. Overall, higher resistance rates were observed among *Enterococcus* isolates, particularly to rifampin (94%), followed by erythromycin and tetracycline (88%, each), and trimethoprim/sulfamethoxazole (54%). Moderate resistance levels were detected for fluoroquinolones, including ciprofloxacin (34%), norfloxacin (32%), and levofloxacin (24%).

**Table 3:** Antimicrobial resistance patterns of *E. faecalis* and *E. faecium* isolates.

AMC	AMA	No. of resistant enterococcus isolates (%)		*p*-value	Total no. of resistant *Enterococcus* isolates (%) (*n* = 50)
		*E. faecalis* (*n* = 27)	*E. faecium* (*n* = 23)		
Penicillins	P	5 (18.5)	3 (13)	0.711	8 (16)
	AM	7 (25.9)	5 (21.7)	0.754	12 (24)
Macrolides	E	23 (85.2)	21 (91.3)	0.674	44 (88)
Tetracyclines	TE	24 (88.9)	20 (87)	1	44 (88)
	DO	5 (18.5)	6 (26.1)	0.733	11 (22)
Fluoroquinolones	CIP	10 (37)	7 (30.4)	0.767	17 (34)
	LEV	7 (25.9)	5 (21.7)	0.754	12 (24)
	NOR	10 (37)	6 (26.1)	0.546	16 (32)
	GAT	4 (14.8)	4 (17.4)	1	8 (10)
Nitrofuran	F	3 (11.1)	3 (13)	1	6 (12)
Rifamycins	RD	26 (96.3)	21 (91.3)	0.588	47 (94)
Phenicols	C	6 (22.2)	6 (26.1)	1	12 (24)
Glycopeptide	VA	2 (7.4)	2 (9)	1	4 (8)
Folate pathway antagonists	SXT	17 (63)	10 (43.5)	0.255	27 (54)

AMA: antimicrobial agent; AMC: antimicrobial class; P: Penicillin; AM: Ampicillin; E: Erythromycin; TE: Tetracycline; DO: Doxycycline; CIP: Ciprofloxacin; LEV: Levofloxacin; NOR: Norfloxacin; GAT: Gatifloxacin; F: Nitrofurantoin; RD: Rifampin; C: Chloramphenicol; VAN: Vancomycin; SXT: Trimethoprim/sulfamethoxazole.

**Table 4:** Antimicrobial resistance patterns of *Enterococcus* isolates from different sources.

AMA	No. of resistant Enterococcus isolates (%)					*p*-value	Total no. of resistant *Enterococcus* isolates (%) (*n* = 50)
	Chicken meat (*n* = 13)	Beef meat (*n* = 15)	Camel meat (*n* = 7)	Workers (*n* = 8)	Consumers (*n* = 7)		
P	2 (15.4)	4 (26.7)	0	1 (12.5)	1 (14.3)	0.635	8 (16)
AM	3 (23.1)	6 (40)	0	1 (12.5)	2 (28.6)	0.299	12 (24)
E	12 (92.3)	14 (93.3)	5 (71.4)	7 (87.5)	6 (85.7)	0.778	44 (88)
TE	11 (84.6)	15 (100)	6 (85.7)	6 (75)	6 (85.7)	0.443	44 (88)
DO	1 (7.7)	4 (26.7)	2 (28.6)	1 (12.5)	3 (42.9)	0.417	11 (22)
CIP	3 (23.1)	7 (46.7)	1 (14.3)	3 (37.5)	3 (42.9)	0.537	17 (34)
LEV	3 (23.1)	6 (40)	0	1 (12.5)	2 (28.6)	0.299	12 (24)
NOR	4 (30.8)	5 (33.3)	0	4 (50)	3 (42.9)	0.323	16 (32)
GAT	3 (23.1)	4 (26.7)	0	0	1 (14.3)	0.363	8 (10)
F	3 (23.1)	2 (13.3)	1 (14.3)	0	0	0.437	6 (12)
RD	11 (84.6)	15 (100)	7 (100)	7 (87.5)	7 (100)	0.284	47 (94)
C	2 (15.4)	5 (33.3)	0	1 (12.5)	4 (57.1)	0.075	12 (24)
VA	0	3 (20)	1 (14.3)	0	0	0.262	4 (8)
SXT	5 (38.5)	8 (53.3)	5 (71.4)	5 (62.5)	4 (57.1)	0.691	27 (54)

AMA: antimicrobial agent; P: Penicillin; AM: Ampicillin; E: Erythromycin; TE: Tetracycline; DO: Doxycycline; CIP: Ciprofloxacin; LEV: Levofloxacin; NOR: Norfloxacin; GAT: Gatifloxacin; F: Nitrofurantoin; RD: Rifampin; C: Chloramphenicol; VAN: Vancomycin; SXT: Trimethoprim/sulfamethoxazole.

In contrast, low resistance rates were observed for vancomycin (8%), followed by penicillin (16%) and gatifloxacin (12%).

Comparison of the two species, no significant variations (*p* > 0.05) were observed in the antimicrobial resistance patterns of *E. faecalis* and *E. faecium* (Table 3). Similarly, no significant differences were observed among the antimicrobial resistance profiles of *Enterococcus* isolates from different sources including meat types, workers, and consumers (Table 4).

**Fig. 5:**
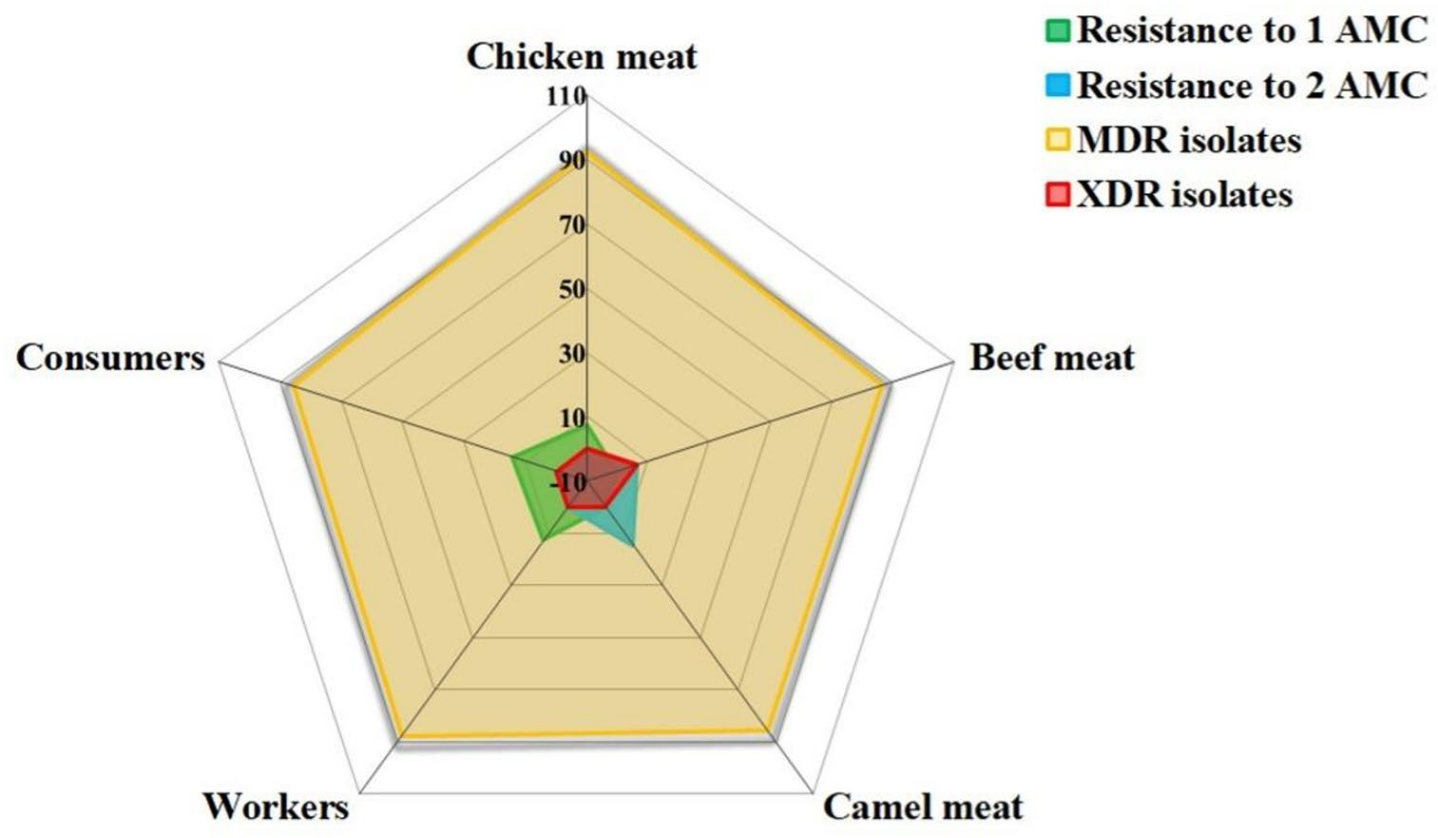
Radar chart showing the occurrence of various resistance categories in *Enterococcus* isolated from different samples. AMC: Antimicrobial classes, MDR: Multiple Drug Resistance, XDR: Extensive Drug resistance.

**Fig. 6:**
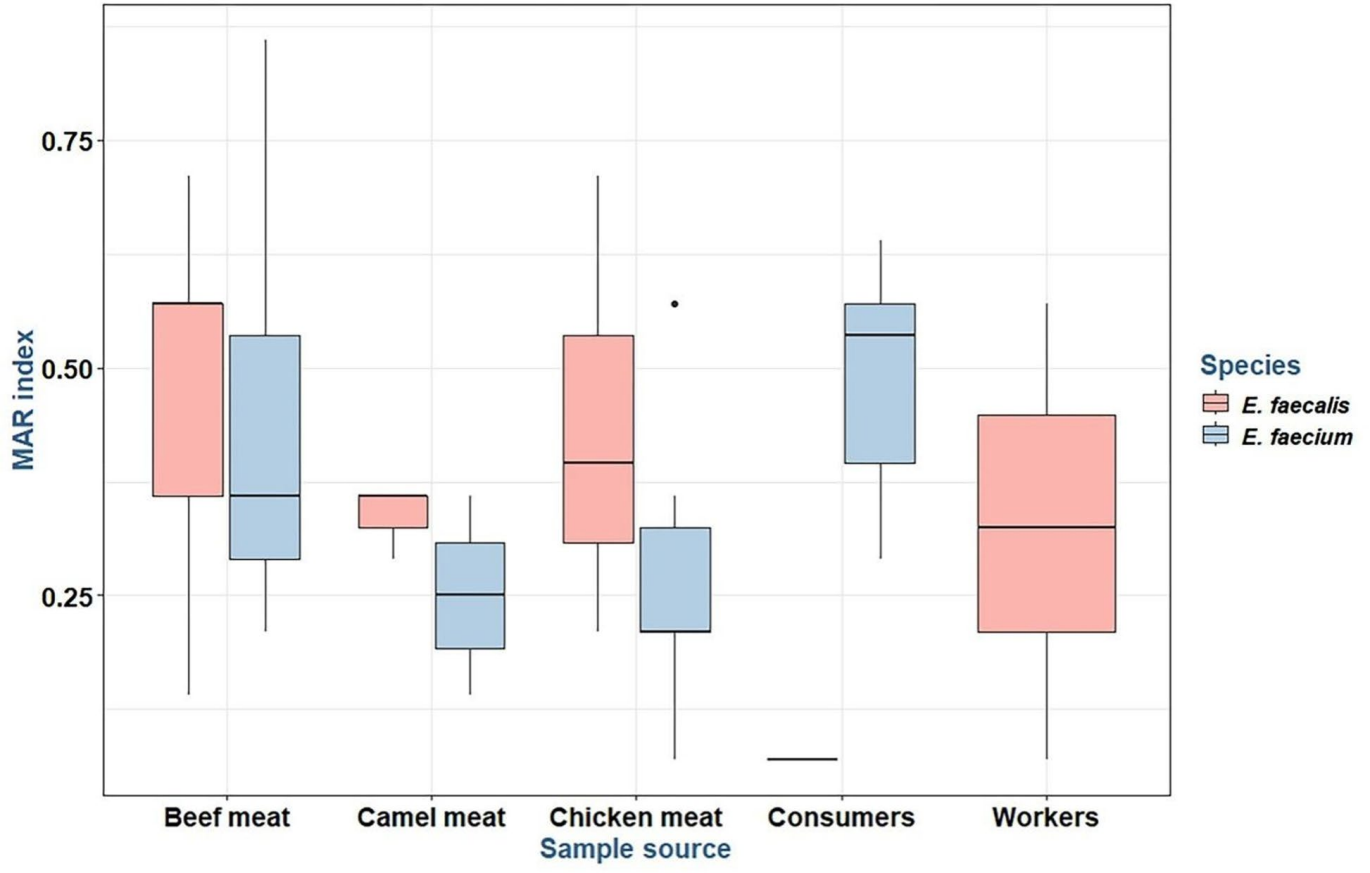
Box plot showing the MAR index of the tested *Enterococcus* isolates belonging to various sources.

Among the 50 *Enterococcus* isolates, 44 (88%) were classified as multidrug-resistant (MDR), while one isolate (2%) was identified as extensively drug resistant (XDR) based on resistance to multiple antimicrobial agents (Table 5; Figs. 5 and 6). The multiple antimicrobial resistance index (MAR) values ranged from 0.07 to 0.86 with a mean value of 0.43.

**Table 5:** Frequency of resistance to various antimicrobial agents in *Enterococcus* isolates belonging to various species.

MARI	Resistant AMA	No. of *Enterococcus* isolates (%)		*p*-value	Total no. of *Enterococcus* isolates (%) (*n* = 50)	Resistance category
		*E. faecalis* (*n* = 27)	*E. faecium* (*n* = 23)			
0.07	1	2 (7.4)	1 (4.3)	1	3 (6)	Resistance to 1 AMA
0.14	2	1 (3.7)	1 (4.3)	1	2 (4)	Resistance to 2 AMA
0.21	3	3 (11.1)	5 (21.7)	0.444	8 (16)	MDR
0.29	4	3 (11.1)	5 (21.7)	0.444	8 (16)	
0.36	5	6 (22.2)	3 (13)	0.479	9 (18)	
0.43	6	3 (11.1)	1 (4.3)	0.614	4 (8)	
0.5	7	1 (3.7)	1 (4.3)	1	2 (4)	
0.57	8	5 (18.5)	4 (17.4)	1	9 (18)	
0.64	9	1 (3.7)	1 (4.3)	1	2 (4)	
0.71	10	2 (7.4)	0	0.493	2 (4)	
0.86	12	0	1 (4.3)	0.46	1 (2)	XDR

AMA: antimicrobial agent; MDR: multidrug-resistant; XDR: extensive drug-resistant.

No significant differences were observed in the prevalence of MAR indices and various resistance categories (*p* > 0.05) among *Enterococcus* isolates from different species, but there was a statistically significant difference in the prevalence of resistance to five antimicrobial classes (*p* = 0.021) among *E. faecalis* and *E. faecium* isolates.

### Biofilm formation ability of *E. faecalis* and *E. faecium* from different sources

**Fig. 7:**
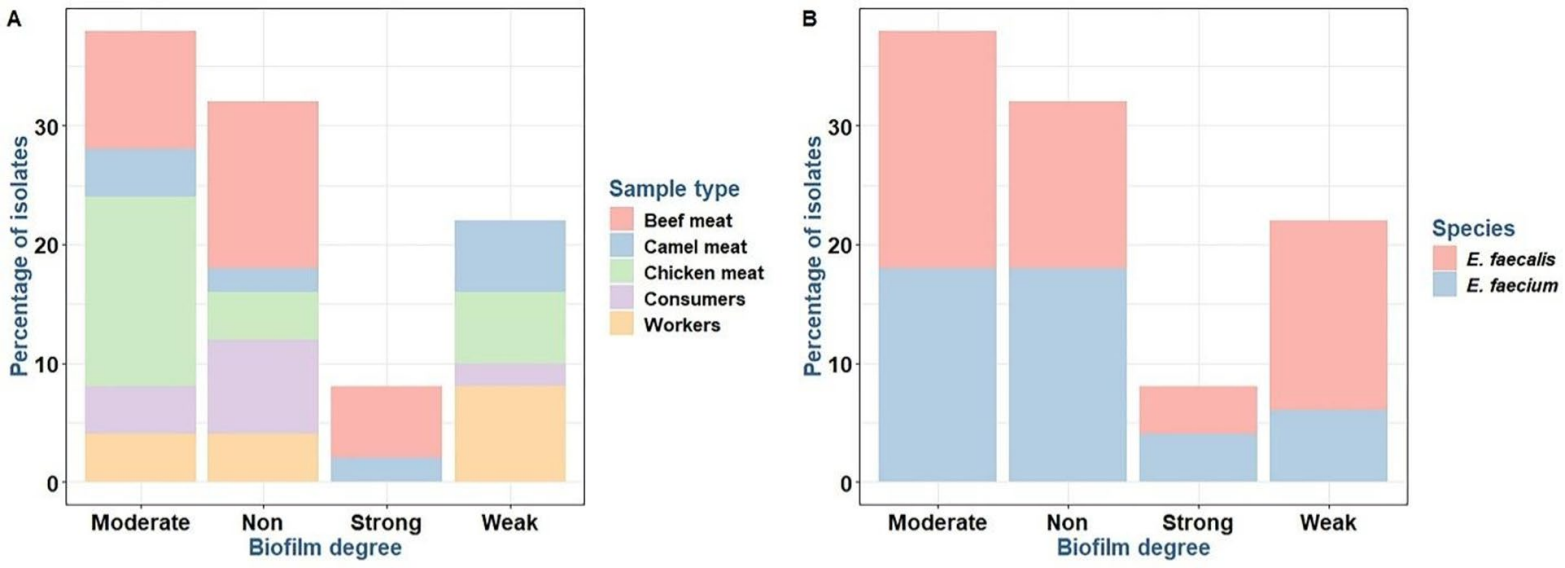
Stacked bar showing the differences in biofilm production abilities between *Enterococcus* isolates belonging to different sources (**A**) and species (**B**). ^*^ The frequency was calculated concerning the total number of the examined isolates (*n* = 50) for each parameter, and subcolumns are calculated as a part of the total column.

The biofilm-forming ability of *Enterococcus* isolates was assessed using the 96 wells microtiter plate assay, the results are illustrated in Table 6; Fig. 7. Moderate biofilm formation was the predominant phenotype (38%, 19/50), followed by non-biofilm producers (32%, 16/50) and weak biofilm producers (22%, 11/50). A small proportion of the isolates (8%, 4/50) were classified as strong biofilm producers.

**Table 6:** Biofilm-forming abilities of *Enterococcus* isolates belonging to different species.

Biofilm production	No. of Enterococcus isolates (%)		*p*-value	Total (*n* = 50)
	*E. faecalis* (*n* = 27)	*E. faecium* (*n* = 23)		
Non	7 (25.9)	9 (39.1)	0.244	16 (32)
Weak	8 (29.6)	3 (13.1)	0.189	11 (22)
Moderate	10 (37)	9 (39.1)	1	19 (38)
Strong	2 (7.4)	2 (8.7)	1	4 (8)

No statistically significant differences were observed in biofilm formation between *E. faecalis* and *E. faecium* in non, moderate, and strong biofilm degrees (*p* > 0.05). However, a significant difference was observed in the distribution of weak biofilm producers among isolates from different sources (*p* = 0.035).

### Resistance and virulence genes profile in *E. faecalis* and *E. faecium* from different sources

**Fig. 8:**
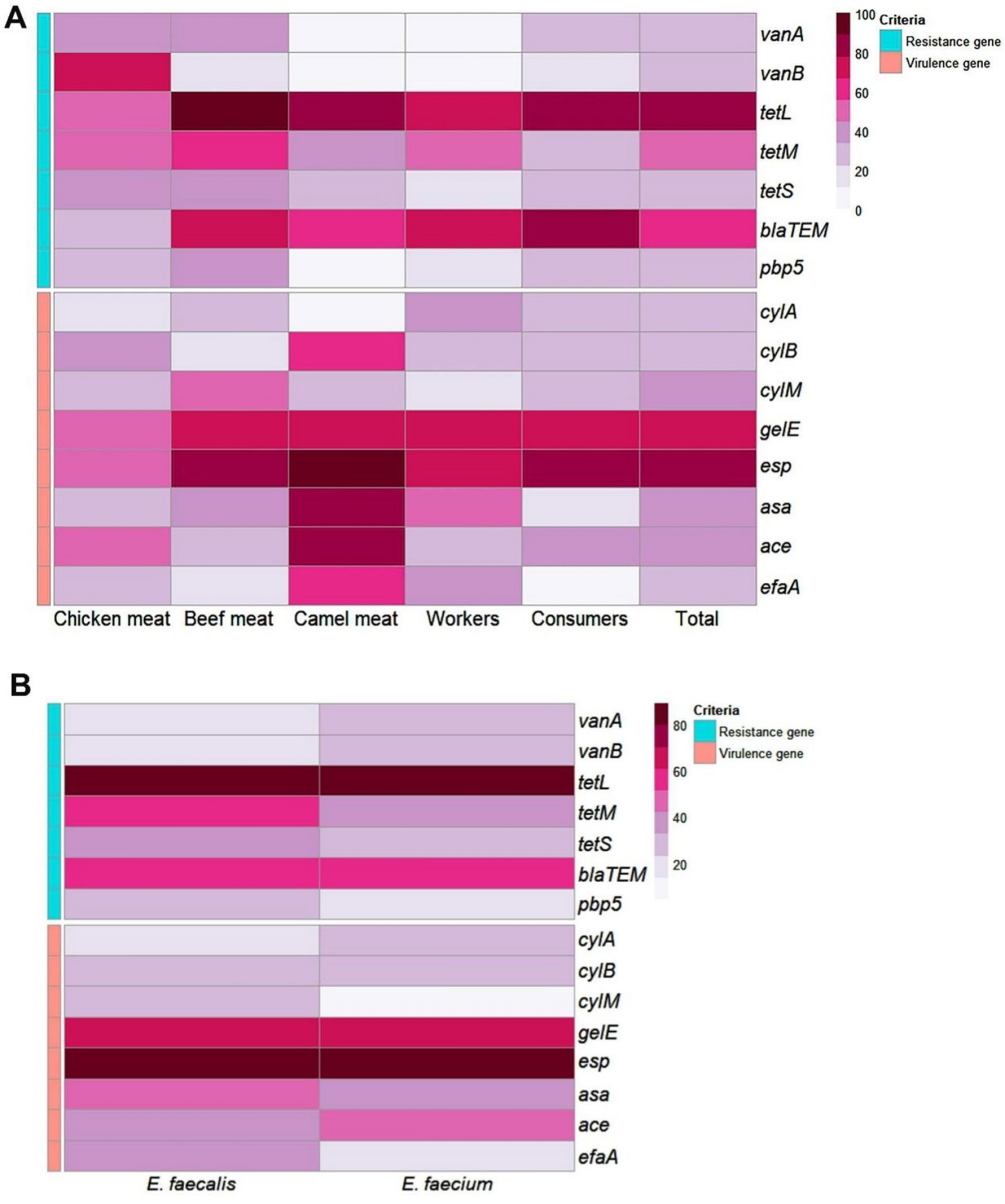
Heatmap showing the prevalence (%) of antimicrobial resistance genes and virulence genes among *Enterococcus* isolates recovered from different sources (**A**), and species (**B**). Color intensity represents the percentages of isolates carrying each gene within the corresponding source or species.

**Fig. 9:**
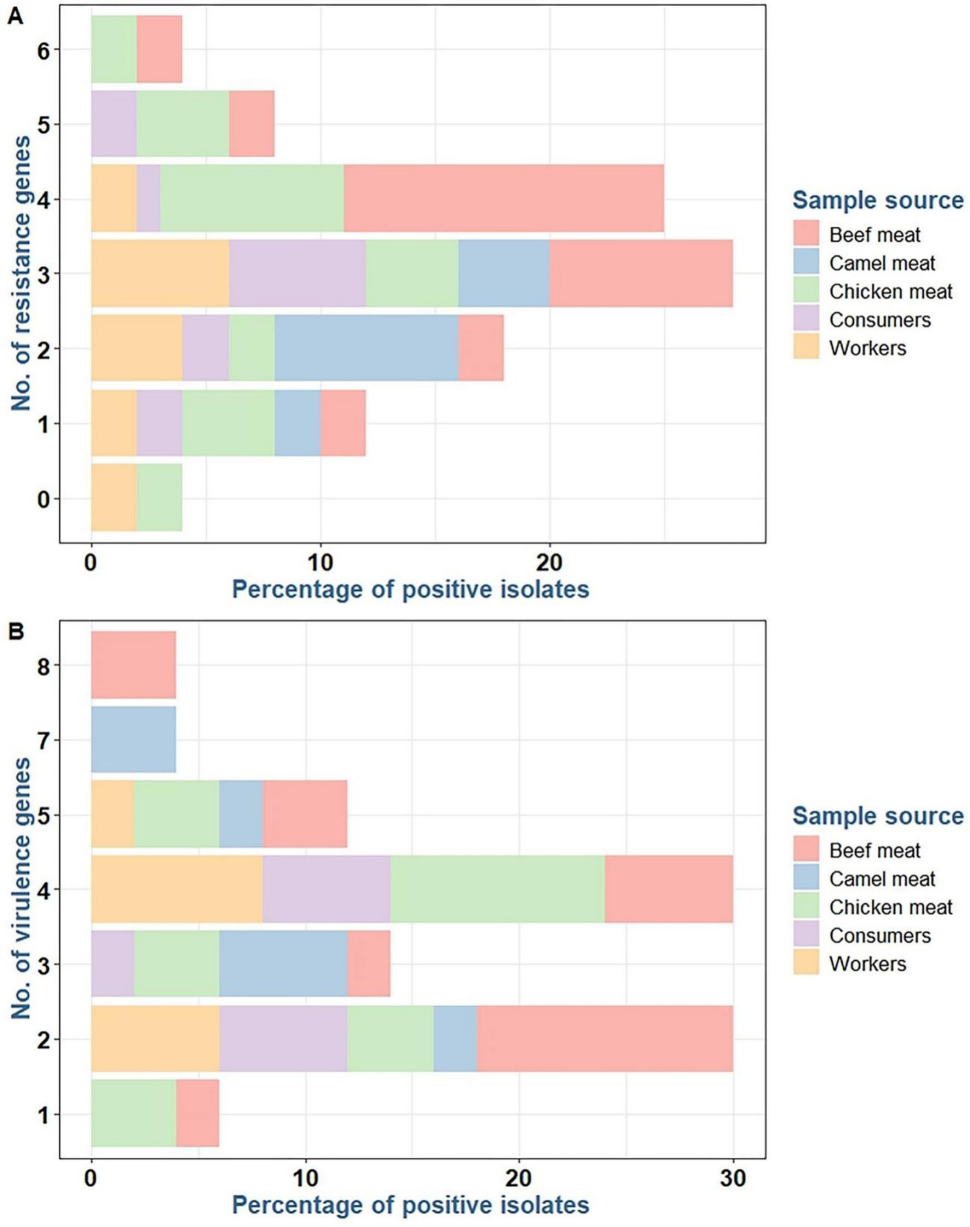
Stacked bar showing the resistance genes (**A**) and virulence genes (**B**) among *Enterococcus* isolates obtained from different sources. ^*^ The frequency was calculated concerning the total number of the examined isolates (*n* = 50) for each parameter, and subcolumns are calculated as a part of the total column.

The distribution of resistance and virulence associated genes profile was determined in *E. faecalis* and *E. faecium* by PCR amplification, the results are illustrated in Table 7; Figs. 3, 8 and 9. Among the antimicrobial resistance genes, *tetL* was the most prevalent (88%, 44/50), followed by *bla*TEM gene (58%, 29/50), *tet*M (48%, 24/50), and *tet*S (32%, 16/50).

**Table 7:** Frequency of resistance genes and virulence genes in *Enterococcus* isolates from different species.

Gene	No. of Enterococcus isolates (%)		*p*-value	Total no. of *Enterococcus* isolates (%) (*n* = 50)
	*E. faecalis* (*n* = 27)	*E. faecium* (*n* = 23)		
Resistance				
* van*A	6 (22.2)	7 (30.4)	0.537	13 (26)
* van*B	5 (18.5)	7 (30.4)	0.508	12 (24)
* tet*L	24 (88.9)	20 (87)	1	44 (88)
* tet*M	16 (59.3)	8 (34.8)	0.098	24 (48)
* tetS*	10 (37)	6 (26.1)	0.546	16 (32)
* bla*TEM	16 (59.3)	13 (56.5)	1	29 (58)
* pbp*5	7 (25.9)	5 (21.7)	0.754	12 (24)
Virulence				
* cyl*A	6 (22.2)	6 (26.1)	1	12 (24)
* cyl*B	8 (29.6)	7 (30.4)	1	15 (30)
* cyl*M	8 (29.6)	9 (4.3)	0.557	17 (34)
* gel*E	18 (66.7)	16 (69.6)	1	34 (68)
* esp*	22 (81.5)	20 (87)	0.711	42 (84)
* asa*	12 (44.4)	8 (34.8)	0.569	20 (40)
* ace*	11 (40.7)	10 (43.5)	1	21 (42)
* efa*A	9 (33.3)	4 (17.4)	0.332	13 (26)

Regarding virulence associated genes, *esp* was the most frequently detected gene (84%, 42/50), followed by *gel*E (68%, 34/50), *ace* (42%, 21/50), and *asa* (40%, 20/50) genes.

Statistically significant differences were observed in the prevalence of *van*B (*p* < 0.0001), *bla*TEM, and *asa* genes (*p* = 0.035 and 0.038, respectively) among *Enterococcus* isolates obtained from human. However, no significant differences were observed in the prevalence of the investigated genes among *Enterococcus* isolates belonging to different species (*p* > 0.05).

### Correlation analysis between phenotypic antimicrobial susceptibility, biofilm-forming ability, resistance genes, and virulence genes

**Fig. 10:**
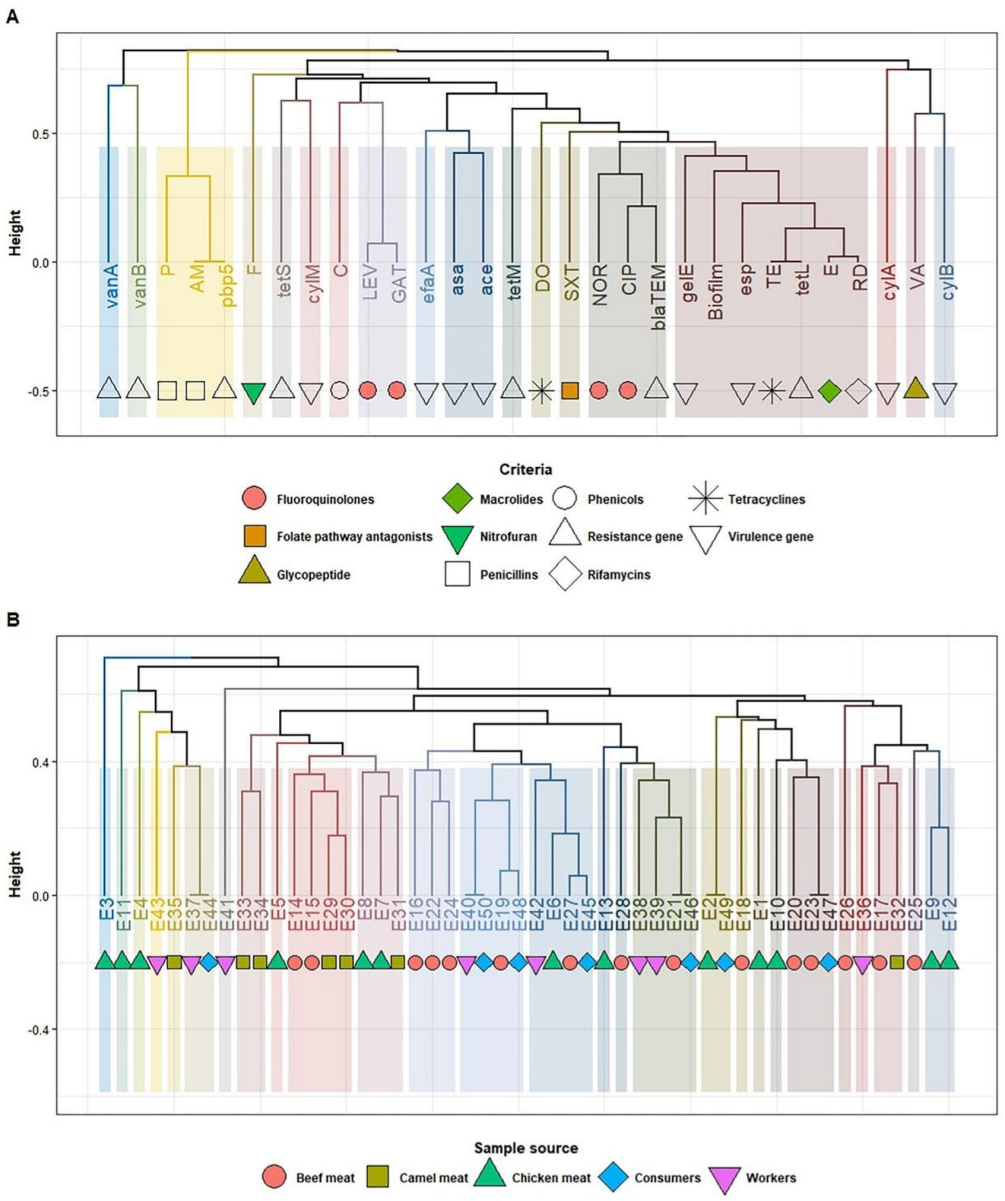
Hierarchical clustering dendrogram displaying the relatedness of the investigated variables (**A**), *Enterococcus* isolates (**B**), as determined by the phenotypic antimicrobial resistance, biofilm production ability, resistance genes, and virulence genes.

**Fig. 11:**
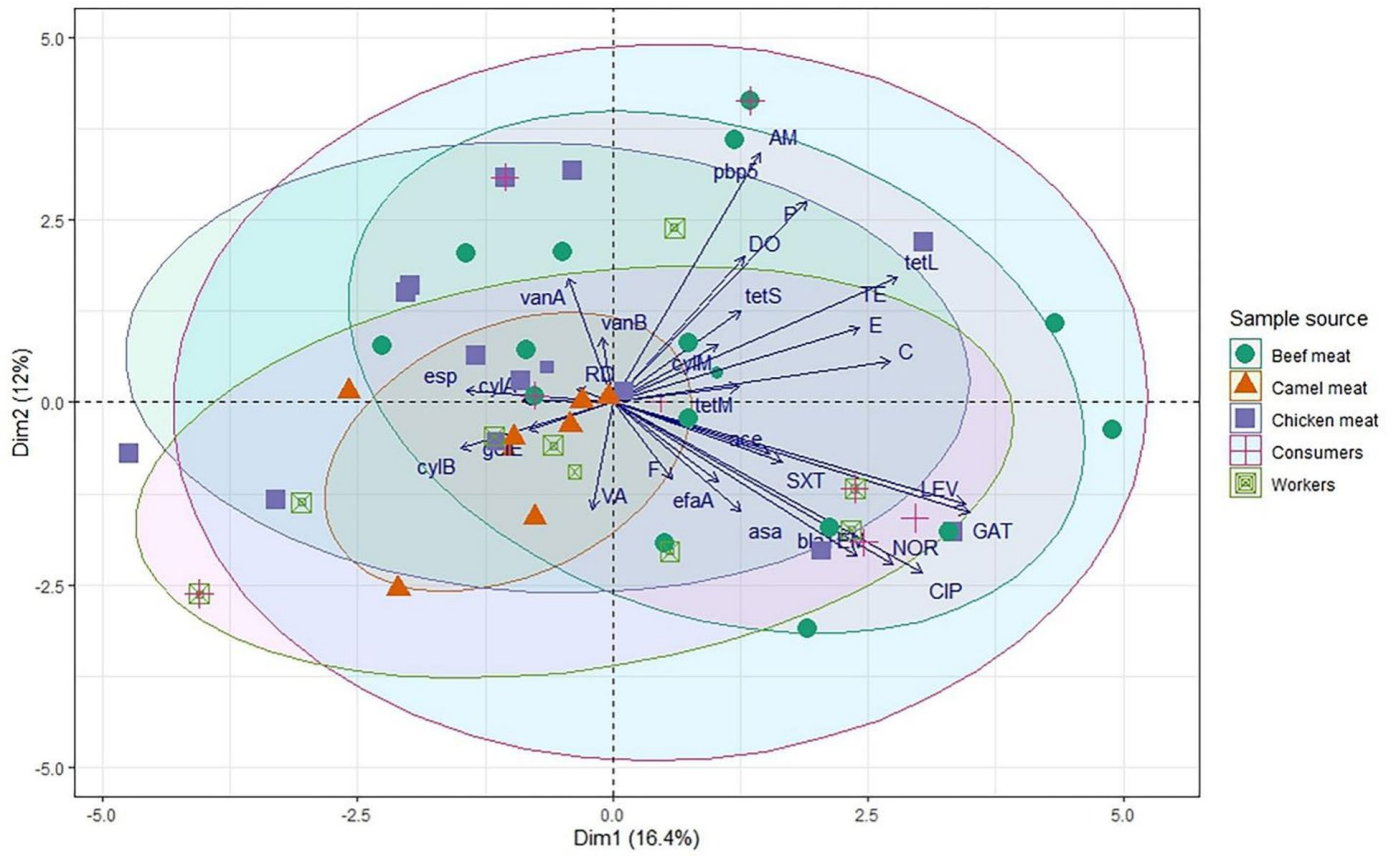
PCA (principal components analysis) biplot of antimicrobial resistance phenotypes, genotypes, virulence genes, and biofilm-forming ability of *Enterococcus* isolates from different sources.

**Fig. 12:**
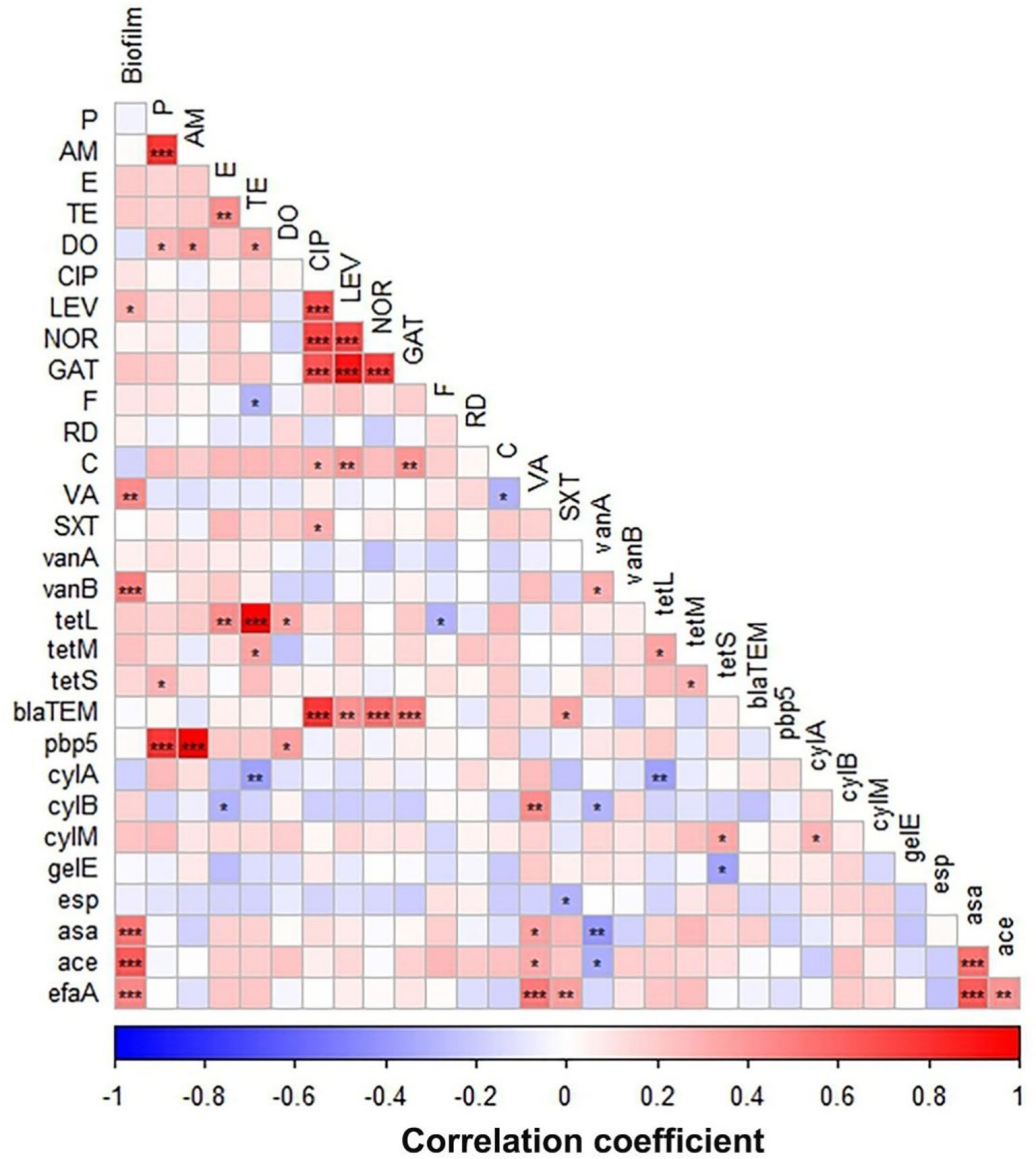
Correlation plot showing the pairwise correlation (*r*) between phenotypic antimicrobial resistance, biofilm production ability, resistance, and virulence genes of Enterococcus isolates from different sources. The scale below the figure refers to the correlation coefficient (*r*). The more intense the color, the more the stronger the positive or negative correlation. Stars refer to the significant correlation; ∗*p* < 0.05, ∗∗*p* < 0.01, ∗∗*p* < 0.001 (details of the correlation coefficient (*r*) are shown in Figure S1).

The relationship among phenotypic antimicrobial resistance, biofilm-forming ability, resistance genes, and virulence genes were analyzed using clustering, principal component analysis (PCA), and correlation analysis (Figs. 10, 11 and 12). Hierarchical clustering analysis revealed a high degree of diversity among the investigated variables, forming 18 branches grouped into two main clusters (Fig. 10A). Similarly, clustering of the *Enterococcus* isolates demonstrated substantial heterogenicity, with 40 out of 50 isolates distributed across distinct lineages, forming 27 branches within five clusters (Fig. 10B). The PCA biplot further supported these findings, showing a non-specific clustering pattern, as isolates belonging to various sources largely overlapped (Fig. 11).

Correlation analysis (Fig. 12) demonstrated several significant associations. A positive correlation was observed between biofilm forming ability and *efa*A, *ace*, and *asa* genes (*r* = 0.47, 0.63, and 0.52, respectively; *p*-value < 0.0001). In addition, biofilm formation was positively correlated with *van*B and resistance to vancomycin (*r* = 0.45, *p*-value < 0.01), and levofloxacin (*r* = 0.29, *p*-value < 0.05). A significant positive correlation was also identified between the *pbp*5 gene and resistance to penicillin, ampicillin, and doxycycline phenotypes (*r* = 0.78, 1, and 0.36, respectively, *p* < 0.05). Conversely, the *cyl*A gene showed a significant negative correlation with both *tet*L and tetracycline resistance phenotype (*r* = − 0.37 each, *p* < 0.01). Similarly, the *cyl*B was negatively correlated with *van*A gene and erythromycin resistance phenotype (*r* = − 0.29 and − 0.3, respectively, *p* < 0.05).

### Genotyping of *E. faecalis* and *E. faecium* from different sources

**Fig. 13:**
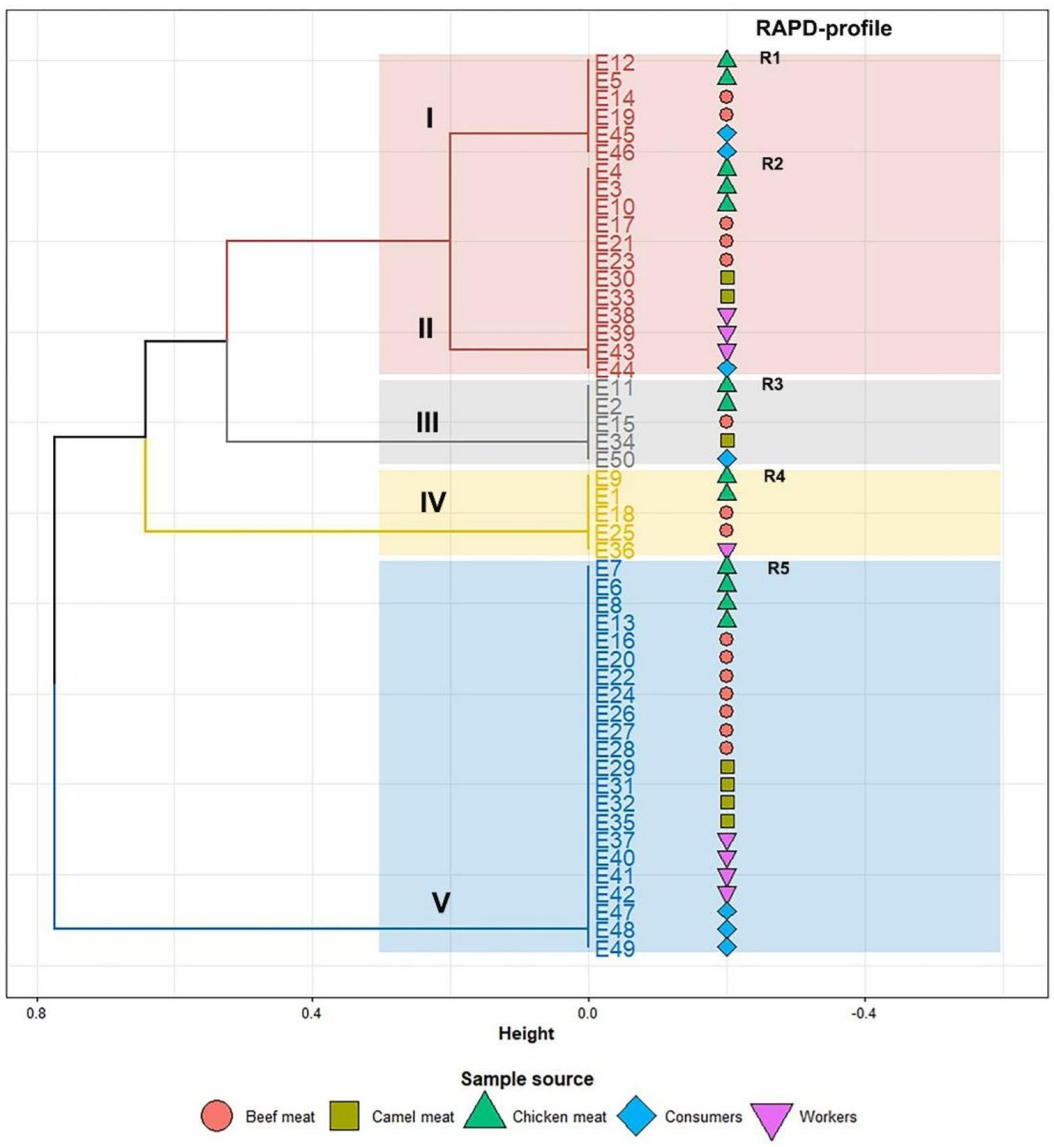
Hierarchical clustering dendrogram showing the relatedness of *Enterococcus* isolates from various samples as determined by RAPD-PCR assay.

The genetic relatedness of *Enterococcus* isolates (*n* = 50) from meat, workers, and consumers were assessed by RAPD-PCR fingerprinting, the results are presented as hierarchical clustering (HA) dendrogram (Figs. 3 and 13). The results revealed five major clusters (I, II, III, IV, and V) comprising four main branches. RAPD analysis identified five distinct RAPD profiles (referred to as R1 to R5) with a moderate discriminatory power (*D* = 0.729). Isolates from different sources, including meat, workers, and consumers, were interspersed within the same clusters and RAPD profiles.

## Discussion

*Enterococcus* species, particularly *E. faecalis* and *E. faecium*, are of significant public health concern due to their role as reservoirs for antimicrobial resistance and virulence determinants. In the present study, both species were recovered from beef, chicken, and camel meat, highlighting the potential role of retail meat as a source for potentially pathogenic *Enterococcus* strains.

*E. faecalis* and *E. faecium* are recognized as opportunistic pathogens in hospital settings, however, they have been isolated from food sources indicating their potential to cause foodborne infection^47^.

The detection of *Enterococcus* species in meat is expected because these bacteria inhabit the animal intestine, emphasizing the importance of hygienic practices during slaughtering and processing of meat^48^. Consequently, *Enterococcus* species are useful indicators of fecal contamination of meat and food products^49^.

The prevalence observed in the present study was lower than that reported in several studies, where isolation rates ranged from 60% to 95% with the predominance of *E. faecalis* and *E. faecium*^47,50,51^. These variations may be attributed to differences in geographic regions, sampling methods, meat types, and hygienic conditions during processing and retail handling.

Although the difference was not statistically significant, *E. faecalis* predominated *E. faecium* in meat samples. This finding is consistent with other reported studies^47,51,52^. The higher prevalence of *E. faecalis* could be attributed to its adaptability under survival and processing conditions^53^.

Regarding meat types, beef samples showed the highest prevalence of *Enterococcus* species in the present study. This is in agreement with other studies reporting variable contamination rates ranging from 53% in South Korea^54^ to 93% in Iran^55^. Chicken meat is also recognized as an important source of *Enterococcus* species, with reported higher isolation rates of 75.5% in United Arab of Emirates^56^ and 28.3% in South Korea^57^. Another study reported a prevalence rate ranged from 28% to 30% during 20 years^58^. In contrast, limited data are available on camel meat, however, the detection of *Enterococcus* species in 14% of camel meat samples in the present study suggests the potential role of camel meat as reservoir of infection. A higher prevalence (40%) was previously reported in Egypt^59^, indicating possible variation due to contamination levels at different geographic areas.

*E. faecalis* and *E. faecium* are opportunistic pathogens capable of causing infections such as urinary tract infections, endocarditis, and sepsis^60^. Their presence in human stool samples reflects their role as intestinal colonizers and reservoirs of pathogenic and antimicrobial-resistant strains. In the present study, *Enterococcus* species were isolated from 15% of human stool samples, with no significant difference between the recovery rate of *E. faecalis* (9%) and *E. faecium* (6%). This prevalence is lower than that reported in Iran (54.9% and 42.1%, respectively)^61^, but higher than that observed in Switzerland (3.8%, each)^62^. Such variability may be attributed to differences in the geographical location, population characteristics, health status of individuals, sampling, and isolation techniques.

The detection of *Enterococcus* species among both workers and consumers, particularly in individuals with diarrhea, indicates their potential role in transmission during food processing stages, leading to dissemination of the pathogen along the food chain^63^. In retail settings, inadequate hygienic practices especially poor hand hygiene, may facilitate the transmission of enterococci from individuals to meat products, and consequently to consumers^64^. Recent studies have reported that *Enterococcus* species can be transmitted to humans through handling and consumption of contaminated food, particularly among occupationally exposed individuals^65^.

In the present study, *Enterococcus* species exhibited significant resistance rates to rifampin, erythromycin, ciprofloxacin, and tetracycline, indicating antimicrobial resistance profile across isolates from meat and human sources. These findings are consistent with elevated resistance of enterococci to macrolides, tetracyclines, and fluoroquinolones^66–68^. The high resistance observed for tetracycline and erythromycin could be attributed to their extensive use in veterinary practice for treatment and prophylaxis, which can promote the selection and persistence of resistance through horizontal gene transfer^50^.

In contrast, low resistance rate to vancomycin was observed in the present study for isolates from meat and human sources, which is consistent with other studies^50,67^. A study in Indonesia reported the absence of vancomycin resistance from *Enterococcus* isolates of chicken meat origin due to the ban of using vancomycin and other glycopeptides in food animals^69^. Global evidence links vancomycin resistance in enterococci to avoparcin use as a growth promoter in animal husbandry suggesting that such resistance patterns may be influenced by historical avoparcin use or by co-selection driven by other antimicrobials^70^. The low resistance rate to vancomycin may reflect the restricted use of glycopeptides in animal production following the ban of avoparcin in many countries. However, the persistence of vancomycin resistant *Enterococcus* isolates in animal husbandry can still be detected because of its long-term viability^57^.

An important finding of the present study was the high prevalence of multi-drug resistance (88%), with most isolates showing MAR index values greater than 0.2, indicating exposure to high-risk sources^50^. The clinical significance of these findings is supported by the classification of MDR *E. faecalis* by World Health Organization (WHO), where the organism as high-priority organisms due to their multidrug resistance and association with healthcare settings^71^. The presence of MDR enterococci in foods of animal origin make them act as reservoirs and transmission vehicles for and antimicrobial resistance (AMR) genes^68,72^. The recovery of MDR enterococci from both meat and human sources supports the hypothesis that these organisms can be transmitted in the food processing chain through handling, contact with the animal carcass, and then to consumers through consumption of meat products^73^. This highlights the importance of implementing strict hygienic measures to limit the spread of resistant enterococci within the One Health framework.

The detection of antimicrobial resistance genes among *Enterococcus* isolates highlights their potential role as reservoirs of transferable resistance determinants within the food chain. High prevalence of resistance genes was observed, with *tet*L (88%) being the most predominant, followed by *bla*TEM (58%), *tet*M (48%), and *tet*S (32%). The predominance of tetracycline resistance genes is consistent with the widespread use of tetracyclines in veterinary practice, which causes selective pressure resulting in the persistence and dissemination of these genes in food producing animals^50^.

In addition, the detection of *van*A (26%) and *van*B (24%) genes among the isolates is of particular health concern, as these genes are commonly associated with vancomycin resistance and are often located in mobile genetic elements, facilitating horizontal gene transfer^58^.

Compared with previous studies, the prevalence of resistance genes observed in the present study appears relatively higher. Lower detection rates were observed for *tet*L gene (10.7%) in beef meat^55^ and *bla*TEM (1.9%) in chicken meat^57^. Moreover, *tet*L and *tet*M genes were identified in beef and chicken meat at retail markets in Nigeria^74^, indicating regional differences in resistance genes distribution. The identification of vancomycin resistance genes in *Enterococcus* isolates is of public health concern due to the potential of transfer from poultry to humans through plasmids and transposons^75^.

*Enterococcus* isolates (68%) exhibited the ability to form biofilms, with moderate biofilm production being the predominant phenotype, followed by weak and strong biofilm producers. This distribution indicates that, although a considerable proportion of the isolates are capable of biofilm formation, the majority exhibit intermediate biofilm-forming capacity, which may reflect the potential of these isolates to persist and survive in food processing environment^70^.

High biofilm forming capacity among *Enterococcus* isolates from food and human sources were also reported^50,76,77^. The comparatively lower proportion of strong biofilm producers in the present study may reflect differences in isolate origin, methods of isolation, or environmental conditions.

Biofilm production associated genes were detected in the present study, *gel*E (68%), *cyl*M (34%), *cyl*B (30%), *efa*A (26%), and *cyl*A (24%). The predominance of *gel*E, a gene encoding gelatinase involved in biofilm development and extracellular matrix formation, is in agreement with Koskeroglu, et al.^50^ who identified *gel*E and *efa*A genes in 52% of the isolates. The high prevalence of these genes indicates the genetic contribution to biofilm production^70^. However, the detection of these genes in moderate and weak producers indicates that gene presence alone is not sufficient to determine biofilm strength. Instead, gene expression levels, environmental conditions, and strain-specific factors are likely to influence the biofilm phenotype^50,70^.

The high frequency of biofilm observed in the present study among isolates from meat and human sources, may contribute to the persistence and dissemination of *Enterococcus* isolates along the food chain. Biofilms enhance bacterial survival under adverse conditions and facilitate the horizontal transfer of antimicrobial resistance and virulence genes, thus increasing the public health risk^76^.

The comprehensive correlation analysis in the present study revealed significant associations between antimicrobial resistance phenotypes, virulence genes and biofilm production. There was a significant positive correlation between biofilm associated genes, antimicrobial resistance, and biofilm phenotypic production. Notably, biofilm forming ability showed a significant positive correlation with *efa*A, ace, and *asa* genes (*r* = 0.47, 0.63, and 0.52, respectively), as well as with *van*B and resistance to vancomycin (*r* = 0.45), and levofloxacin (*r* = 0.29). These findings indicate that isolates with enhanced adhesion capabilities are more likely to exhibit increased antimicrobial resistance. Furthermore, a significant positive correlation was observed between the *pbp*5 gene and resistance to penicillin, ampicillin, and doxycycline phenotypes (*r* = 0.78, 1, and 0.36, respectively) indicating the potential role of this gene in mediating resistance to β-lactam antibiotics.

The observed positive associations between biofilm formation, antimicrobial resistance, and virulence genes are consistent with other reported studies^50,76,78^. The acquisition of specific antimicrobial resistance can enhance biofilm formation in several species of Gram-negative bacteria^78^. Such co-occurrence might be facilitated by horizontal gene transfer events within biofilms, which facilitate the exchange of resistance determinants among sessile cells^50,79^. In contrast, other studies reported that isolates with high resistance to antimicrobials produced weak biofilms^80^, however, they still provide a level of protection of the isolates^76^.

The genetic relatedness of *E. faecalis* and *E. faecium* isolates from meat and human sources was evaluated by RAPD-PCR fingerprinting. Five major clusters (I-V) and five distinct RAPD profiles (R1-R5) were identified. The discriminatory power of the RAPD-PCR was moderate with a *D* value of 0.729 indicating a reasonable ability of the method to differentiate isolates. Isolates derived from meat, retail workers, and consumers were present in the same clusters, indicating a high degree of genetic relatedness across different sources, which may suggest a potential of cross-transmission^81^.

The moderate discriminatory power observed in this study is comparable with previous investigations demonstrating the usefulness of RAPD-PCR to effectively reveal genetic diversity among *Enterococcus* isolates and its applicability in epidemiological investigations and tracing sources^82,83^ The clustering pattern observed in the present study, especially the lack of clear distinctions between human and meat isolates, indicates that genetically related *Enterococcus* strains can be shared between isolates from animal and human sources^81^. The intermixing of isolates from different sources within the same clusters may indicate a potential cross transmission between food and human reservoirs.

The integration of phenotypic, genotypic, and clustering analysis in the present study provides important insights into the potential co-selection and dissemination of antimicrobial resistance and virulence traits among *Enterococcus* species. The observed associations between biofilm formation, resistance phenotypes, and virulence genes together with the genetic relatedness between isolates from meat and human sources, support the hypothesis that the food chain may play a key role in the transfer of antimicrobial-resistant *Enterococcus* strains across the food-human interface.

## Conclusions

The present study shows that *Enterococcus* species, particularly *E. faecalis* and *E. faecium* isolated from retail meat, workers, and consumers, represent a significant public health concern. A considerable proportion of the isolates were multi-drug resistant, and virulence associated genes were identified highlights the potential pathogenicity of the isolates. Correlation analysis revealed significant associations between antimicrobial resistance, biofilm formation, and virulence genes suggesting possible co-selection and enhanced adaptability. RAPD-PCR fingerprinting indicated genetic relatedness among the isolates from meat and human sources, suggesting potential epidemiological link in the food chain. Improved hygiene practicies during meat handling, especially strict hand hygiene among workers, are crucial to minimize cross-contamination. Routine monitoring of antimicrobial resistance in food-producing animals and retail meat, along with awareness programs for food handlers and consumers, may help to reduce the dissemination of multidrug-resistant *Enterococcus* species within the food chain.

### Limitations

A limitation of this study is the use of convenience sampling, which may affect the generalization of the findings. Future studies using large, randomized sampling approaches are recommended. Furthermore, the cross-sectional design of the study, the limited number of isolates, and the use of RAPD-PCR do not allow confirmation of direct transmission pathways. Therefore high-resolution methods such as whole genome sequencing (WGS) are required to confirm transmission dynamics in future studies.

## Data Availability

All data generated or analyzed during this study are included in this published article. Gene amplification was performed for confirmation of target genes using previously published primers. No novel gene sequences were generated in this study.
